# Virtual Resection: A New Tool for Preparing for Nephron-Sparing Surgery in Wilms Tumor Patients

**DOI:** 10.3390/curroncol29020066

**Published:** 2022-02-01

**Authors:** Jasper M. van der Zee, Matthijs Fitski, Frank F. J. Simonis, Cornelis P. van de Ven, Aart J. Klijn, Marc H. W. A. Wijnen, Alida F. W. van der Steeg

**Affiliations:** 1 Department for Pediatric Surgery, Princess Máxima Center for Pediatric Oncology, 3584 CS Utrecht, The Netherlands; zeejasper96@gmail.com (J.M.v.d.Z.); m.fitski-2@prinsesmaximacentrum.nl (M.F.); c.p.vandeven-4@prinsesmaximacentrum.nl (C.P.v.d.V.); m.h.w.wijnen-5@prinsesmaximacentrum.nl (M.H.W.A.W.); 2 Technical Medicine, TechMed Centre, University of Twente, 7522 NB Enschede, The Netherlands; 3 Magnetic Detection & Imaging, TechMed Centre, University of Twente, 7522 NB Enschede, The Netherlands; f.f.j.simonis@utwente.nl; 4 Department of Pediatric Urology, University Medical Center Utrecht/Wilhelmina Children’s Hospital, 3584 CX Utrecht, The Netherlands; a.j.klijn@umcutrecht.nl

**Keywords:** Wilms tumor, virtual resection, remnant renal volume, nephron-sparing surgery, partial nephrectomy, remnant renal parenchyma

## Abstract

Nephron-sparing surgery (NSS) in Wilms tumor (WT) patients is a surgically challenging procedure used in highly selective cases only. Virtual resections can be used for preoperative planning of NSS to estimate the remnant renal volume (RRV) and to virtually mimic radical tumor resection. In this single-center evaluation study, virtual resection for NSS planning and the user experience were evaluated. Virtual resection was performed in nine WT patient cases by two pediatric surgeons and one pediatric urologist. Pre- and postoperative MRI scans were used for 3D visualization. The virtual RRV was acquired after performing virtual resection and a questionnaire was used to assess the ease of use. The actual RRV was derived from the postoperative 3D visualization and compared with the derived virtual RRV. Virtual resection resulted in virtual RRVs that matched nearly perfectly with the actual RRVs. According to the questionnaire, virtual resection appeared to be straightforward and was not considered to be difficult. This study demonstrated the potential of virtual resection as a new planning tool to estimate the RRV after NSS in WT patients. Future research should further evaluate the clinical relevance of virtual resection by relating it to surgical outcome.

## 1. Introduction

Wilms tumor (WT), also known as nephroblastoma, is the most frequently occurring renal tumor in children, with a five-year survival rate of ~90% [1,2,3]. Approximately 35 children are diagnosed with WT in the Netherlands annually, and in most cases this is a unilateral tumor. In 5–10% of WT patients, the disease is bilateral with an increased likelihood for end-stage renal disease and secondary morbidity [4]. Treatment of WT is in accordance with the UMBRELLA treatment protocol prescribed by the Renal Tumor Study Group of the International Society of Pediatric Oncology (SIOP-RTSG) [5]. This treatment protocol describes neoadjuvant chemotherapy, followed by open radical or partial nephrectomy, also known as nephron-sparing surgery (NSS), and adjuvant chemotherapy. The preferred surgical treatment in bilateral and syndromic unilateral patients is NSS with radical resection of the tumor to preserve as much functional remnant renal volume (RRV) as possible.

In nonsyndromic patients, radical nephrectomy is the standard of care, and NSS is limited to certain patients who meet the criteria established in the SIOP-RTSG UMBRELLA treatment protocol 2016. These criteria should prevent worse oncological outcome due to irradical resection (R1 or R2) that upstages the tumor and implies the addition of radiotherapy [6]. However, NSS may reduce the risk of end-stage renal failure and allow for more surgical treatment options in case of a metachronous tumor in the contralateral kidney [7]. NSS cases require extensive preoperative planning to ensure a safe oncological outcome and the preservation of functional RRV.

For the preoperative planning of NSS, three-dimensional (3D) visualization is routinely used in the Princess Maxima Center. The introduction of this technique improved the anatomical orientation of surgeons performing oncologic renal surgery [8,9,10,11]. In addition, Isotani et al. showed that 3D visualizations could be used for virtual resection of renal tumors in adults [12]. This technique allows surgeons to virtually perform NSS and estimate the RRV preoperatively. However, this technique has not yet been implemented in pediatric oncologic surgery. In this study, a method for virtual resection planning of NSS for WT patients and the user experience of virtual resection are evaluated by the surgeons.

## 2. Materials and Methods

In this single-center study, the feasibility of virtual resection was examined as an additional tool for preoperative NSS planning for WT patients using retrospective acquired imaging data. Three dimensional visualizations were prepared with the in-house developed 3D imaging workflow for NSS developed by Fitski et al. [13]. Additionally, the actual RRV of the patient was computed after 3D visualization of the available postoperative magnetic resonance imaging (MRI) scans. Secondly, virtual resections were performed by two pediatric surgeons and one pediatric urologist. Thirdly, the derived virtual RRVs were compared with the actual RRV, resulting in a volume fraction. Finally, surgeons were asked to complete a questionnaire to assess the user experience of virtual resection in terms of technical performance and clinical relevance.

### 2.1. Patient Inclusion

This study was performed using retrospective imaging data of WT patients who underwent NSS and received both a pre- and postoperative MRI in the Princess Maxima Center in The Netherlands between 01/01/2019 and 01/07/2021. All NSS patients received standard care in accordance with the SIOP-RTSG UMBRELLA treatment protocol 2016. Within this protocol, patients received preoperative MRI and if the tumor was pathologically characterized as high risk, postoperative MRI was also performed. Twelve patients were considered for NSS during this period. In six patients, the tumor was pathologically characterized as high risk, and postoperative MRI was performed. Of these six patients, three had surgery on both kidneys, which resulted in nine single operative cases. The Institutional Ethics Review Board waived the necessity of informed consent since the study did not involve the actual patients and treatment was not influenced. All patients were included in the UMBRELLA protocol and signed the UMBRELLA patient information form.

### 2.2. Imaging and 3D-Visualization

All patients were scanned, under sedation, with a 1.5 tesla MRI system (Achieva, Philips Medical Systems, Best, The Netherlands). In addition, 3D visualizations were performed with the acquired MRI scans in the 3D Slicer (version: 4.11.20210226) software package [14]. To determine the actual RRV, a post-contrast fat-suppressed T1-weighted MRI sequence was used in accordance with the visualization protocol developed by Fitski et al. [13].

### 2.3. Virtual Resection

Virtual resection was performed by two pediatric surgeons and one pediatric urologist with extensive experience in NSS. For the virtual resection, an open-source extension was used in 3D Slicer: ResectionPlanner. To gain familiarity with the system and virtual resection, the surgeons performed a training case.

**Figure 1:**
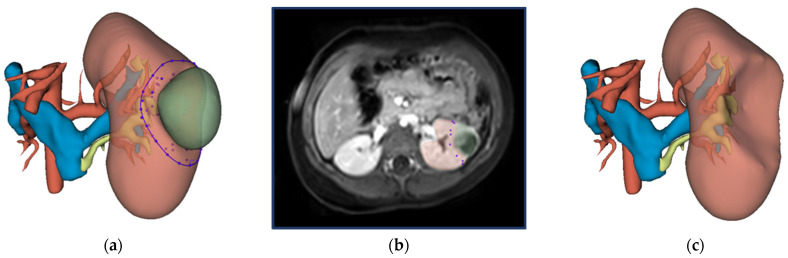
Workflow for virtual resection in 3D Slicer: (**a**) 3D visualization of the kidney, tumor, urinary collecting system, renal artery and renal veins. The closed curve as selected by the surgeon is indicated in purple. (**b**) Preoperative MRI imaging (post-contrast fat-suppressed T1-weighted) of the abdomen with the kidney and WT segmentation in red and green, respectively. The surgeon selected intraparenchymal points on this MRI scan. (**c**) 3D visualization of the virtual kidney volume after virtual resection.

The surgical protocol for NSS consists of identifying the tumor with intraoperative ultrasound, followed by circumscribing the resection border with diathermy, and subsequent radical tumor removal [15]. The virtual resection was designed to mimic this surgical approach. The methodology for virtual resection is visualized in Figure 1. The surgeon was able to gain familiarity with the patient’s anatomy by inspecting the 3D visualization and the available imaging data beforehand. After inspection, resection started with the surgeon selecting several points on the surface of the kidney and the resection software computed a closed curve between these points. This closed curve is visualized with the purple line in Figure 1a and represents the circumscription of the resection border with diathermy. Secondly, the surgeon selected several intraparenchymal points in the available imaging data (shown in Figure 1b). Both the closed circle and intraparenchymal points were combined and used as input for the ResectionPlanner. This resulted in a 3D model of the virtual remnant kidney used for the computation of the virtual RRV shown in Figure 1c. Finally, the surgeon was able to perform small final corrections on the 3D model with tools available in 3D Slicer.

### 2.4. Volumetric Assessment

The performance of virtual resection was evaluated with the agreement of the virtual RRV and the actual RRV by computing a volume fraction. The volume fraction was computed by dividing the virtual postoperative kidney volume by the actual postoperative kidney volume (Equation (1)). Ideally, the virtual resection volume matches perfectly with the actual postoperative volume resulting in a volume fraction of 1.0. A volume fraction >1.0, implies less volume was resected by virtual resection than during the actual surgery, and thus the virtual RRV was overestimated compared with the actual RRV. Underestimation, volume fraction < 1.0, implies more volume was resected by virtual resection than during the actual surgery.

(1) $$Volume\;fraction=\frac{V_{postoperative,\;\;\;virtual}}{V_{postoperative,\;\;\;actual}}\;$$

### 2.5. User Experience

The surgeons were asked to complete an in-house developed questionnaire. Each statement was scored on a Likert-scale from 1 to 5 ranging from ’strongly disagree’ (1) to ‘strongly agree’ (5). The questionnaire contained six statements. Two of the statements measured the technical performance as experienced by the surgeon: S1 and S4. Four of the statements evaluated the clinical relevance: S2, S3, S5 and S6.

### 2.6. Statistics

All statistical analyses were performed using SPSS Statistics Version 27 (IBM Corp., Armonk, NY, USA). For the volumetric assessment, the median and the interquartile range (IQR) were computed. For the user experience analysis, answers per statement per surgeon were collected and the median and the interquartile range (IQR) were determined.

## 3. Results

### 3.1. Patient Characteristics

The six patients had a mean age of 48 months (STD = 32 months). The complete preoperative 3D visualization was successfully obtained with MRI data only in 7 of the 9 cases. In cases eight and nine, 3D visualization of the kidney and tumor were obtained from preoperative MRI. However, the vascular system and the urinary collecting system (UCS) were obtained from computed tomography. Subsequently, the 3D models were accurate, manually matched with the 3D models derived from the preoperative MRI. Patient demographics, tumor characteristics and the time between NSS and acquisition of the postoperative scan are described in Table 1.

### 3.2. Volumetric Assessment

**Figure 2:**
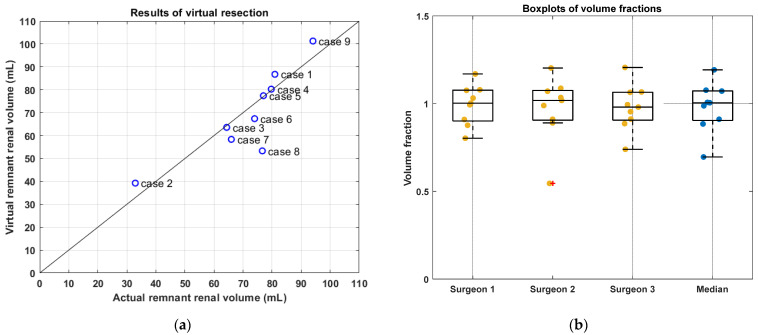
Results of virtual resection. In (**a**), the average virtual and the actual postoperative volume per case are shown. The black line implies a volume fraction equal to one, which corresponds to perfect agreement between the virtual and actual postoperative volume. In (**b**), the volume fraction per case is shown per surgeon and the median.

Radical tumor resection was performed in all cases in both the actual and virtual resection. The actual and virtual postoperative volumes are visualized in Figure 2a. Most of the results are located near the black line which implies a volume fraction equal to one. In case eight, the tumor volume was three times larger than the kidney volume and a large resection was required. For this large resection, minor deviations in the surgical approach by the different surgeons caused a large difference in RRV among the surgeons and a relatively low volume fraction. In case nine, four additional tumor resections were performed next to the two tumors that were seen in preoperative MRI scans. This resulted in an overestimation of the virtual RRV. The volume fractions derived by each surgeon are visualized in Figure 2b and shown in Table 2. Based on the RRVs given in Table 2, the agreement among observers appears acceptable. The median volume fraction was found to be 0.94 (IQR = 0.16).

### 3.3. User Experience

The results of the questionnaire are summarized in Table 3. Virtual resection was not considered difficult and the surgeons found virtual resection straightforward. No clear opinion was derived for the usefulness of the derived line of resection in the intraoperative decision-making. There was a large variation between surgeons on whether the real-life surgical tumor resection was considered difficult.

## 4. Discussion

This study evaluated virtual resection as a novel method to mimic tumor resection and estimate the RRV in WT patients. With virtual resection, surgeons can estimate the postoperative RRV with a nearly perfect matching volume fraction. Moreover, surgeons found the technique straightforward and not difficult. These features allow implementation in the current NSS planning to be feasible.

Comparable work of virtual resection in renal malignancies has been conducted in adults. Isotani et al. showed a significant correlation between the actual RRV and the virtual RRV based on the postoperative weight of the specimen [12]. Using the volume of the specimen, instead of the postoperative MRI that is not routinely performed in every patient, allows for the inclusion of more patients in further prospective research. Ueno et al. showed that virtual resection allowed for accurate estimation as to whether the UCS had to be opened [16]. The addition of the UCS in 3D visualizations could improve the orientation of critical anatomical structures of virtual resection and therewith the clinical relevance of virtual resection.

Intraoperative decisions may deviate from the planned resection based on preoperative imaging. Such differences between the virtual resection planning and the actual performed surgery were found in several cases. In case 9, six lesions were found intraoperatively and resected, of which four lesions were not visible on preoperative imaging and therefore not included in virtual resection. Apparently, not all lesions appear visible in preoperative MRI scans that result in a deviation of the planned resection. In cases 6 and 7, an increase in the actual postoperative renal volume was found in comparison with the actual preoperative renal volume, suggesting postoperative growth of the kidney. Postoperative growth can be explained by hypertrophy because of postoperative adaptations in the kidney [17]. In addition, postoperative hydronephrosis may also contribute to the increase in postoperative renal volume. To correct for postoperative growth, comparison with the contralateral kidney volume may allow for a more accurate estimation of the actual RRV. This may further improve the validation of virtual resection.

The clinical relevance of virtual resection must be evaluated before virtual resection influences the surgical approach in pre- and intraoperative decision-making and is implemented in current NSS planning. In this study, using the closed curve on the kidney’s surface was found to be clinically relevant by one of the surgeons. This surgeon reported that intraoperative circumscription of the tumor would be less complicated after determining the closed curve virtually. Nevertheless, results from the questionnaires showed that the clinical relevance of virtual resection for these nine cases was deemed limited as all surgeons were familiar with all cases. In further research, virtual resection needs to be performed before the actual surgery to fully assess its clinical value on intraoperative decisions by pediatric surgeons. The clinical usability of virtual resection may be improved by adding more estimation features than solely the reduction in renal volume. Correlating renal function and RRV may result in a more accurate estimation of postoperative outcome than RRV alone [18,19]. Secondly, virtual resections can be used to predict possible surgical complications such as urine leakage or a positive surgical resection margin [20]. Based on virtual resection, the postoperative RRV can now be estimated, which can be used for the indication of hemodialysis or chronic peritoneal dialysis catheters. Moreover, virtual resection provides an estimation of the opening of the UCS and therefore the indication for a double J catheter [12,16]. Thus, knowledge of the expected RRV and postoperative renal function, next to the preoperative clinical status, can influence decisions concerning the indication of dialysis catheters during NSS for acute renal failure.

This study has some limitations that need to be acknowledged. This was a single-center study using retrospective acquired imaging data of patients that already underwent NSS and thus the surgeons were familiar with all of the cases. Therefore, the clinical relevance rated by the surgeons could be affected. Second, only a limited number of cases were available for inclusion due to the requirements of both pre- and postoperative MRI. More patients can be included in further research when using a volumetric assessment of pathological specimen instead of the limited available postoperative MRI [12]. Further research of virtual resection in combination with renal function and surgical complications is required to validate and strengthen the clinical relevance of this potential new tool for NSS planning.

## 5. Conclusions

This study demonstrated the potential of virtual resection as a new planning tool to estimate the RRV after NSS in WT patients. Virtual resection appeared to be a straightforward technique that is not difficult to use, hence implementing virtual resection in current NSS preoperative planning seems feasible. Future research should evaluate the added clinical value of simulating tumor resection during preoperative planning and incorporating surgical outcome, such as renal function and the indication for hemodialysis or chronic peritoneal dialysis catheters, additional to estimating RRV, to further validate and strengthen the clinical relevance of virtual resection as a new tool in NSS planning.

## Figures and Tables

**Table 1:** The patient demographics, tumor characteristics and the time between the scans and surgery are listed for the nine operative cases.

Variable	Case 1 *	Case 2	Case 3	Case 4	Case 5 *	Case 6 ^‡^	Case 7 ^‡^	Case 8 ^ψ^	Case 9 ^ψ^
Gender (M/F)	F	M	F	F	F	M	M	M	M
Age (months)	106	14	41	40	106	30	30	54	54
Disease	UF	UF	UF	UF	UF	UF	UF	MF	UF
Location	Left	Left	Left	Right	Left	Left	Right	Left	Right
Position	LP	UP	MP	MP	UP	LP	LP	LP and MP	MP
Syndrome	-	BWS	-	WT-1	-	-	-	BWS	BWS
The time between NSS and acquisition of the postoperative scan (days)	20	187	65	126	35	48	48	386	48

M = male, F = female, UF = unifocal, MF = multifocal, UP = upper pole, MP = mid pole, LP = lower pole, BWS = Beckwith–Wiedemann Syndrome, WT-1 = Wilms tumor 1 mutation. The superscripts (*, ^‡^ and ^ψ^) imply the same patient resulting in two single operative cases.

**Table 2:** The RRVs per surgeon for all nine operative cases.

Case Number	Surgeon 1	Surgeon 2	Surgeon 3
1	97.4	98.2	96.8
2	94.5	91.9	94.8
3	91.7	92.1	90.8
4	100.0	99.7	92.2
5	98.2	96.7	95.9
6	99.9	99.7	100.0
7	99.5	98.0	99.0
8	34.4	50.7	46.7
9	94.6	93.5	92.7

**Table 3:** Results of the questionnaire were filled in by each surgeon. The results represent the patient cumulative opinion per statement. The table visualizes both the opinion per clinician in addition to the median outcome.

	Statement	Median	IQR
1.	The virtual resection as performed in 3D Slicer was straightforward.	4.0	1.5
2.	The derived line of resection, as created in 3D Slicer is useful in the intraoperative decision-making.	3.0	1
3.	This virtual resection gives a better insight into other critical anatomical structures in addition to the standard preoperative 3D planning.	3.0	1
4.	I classify this virtual resection, as performed in 3D Slicer, to be difficult.	1.0	1.5
5.	Virtual resection, as performed according to this protocol, affects my intraoperative decision.	2.0	1
6.	I expect this real-life surgical tumor resection, in this particular case, to be difficult.	2.0	2.5

## Data Availability

The data presented in this study are available on request from the corresponding author.
